# The Effect of Commercial Activity Tracker Based Physical Activity Intervention on Body Composition and Cardiometabolic Health Among Recent Retirees

**DOI:** 10.3389/fragi.2021.757080

**Published:** 2021-10-29

**Authors:** Tuija Leskinen, Kristin Suorsa, Ilkka HA Heinonen, Eliisa Löyttyniemi, Jaana Pentti, Jussi Vahtera, Sari Stenholm

**Affiliations:** ^1^ Department of Public Health, University of Turku and Turku University Hospital, Turku, Finland; ^2^ Centre for Population Health Research, University of Turku and Turku University Hospital, Turku, Finland; ^3^ Turku PET Centre, and Department of Clinical Physiology and Nuclear Medicine, University of Turku, Turku, Finland; ^4^ Rydberg Laboratory of Applied Sciences, Department of Environmental- and Biosciences, University of Halmstad, Halmstad, Sweden; ^5^ Department of Biostatistics, University of Turku and Turku University Hospital, Turku, Finland; ^6^ Clinicum, Faculty of Medicine, University of Helsinki, Turku, Finland

**Keywords:** activity tracker, physical activity, metabolism, body composition, randomized controlled trial, older adults

## Abstract

The REACT is a commercial activity tracker based intervention, which primarily aimed to increase physical activity. This study examines the secondary outcomes of the physical activity intervention on body composition and cardiometabolic health indicators. Overall 231 recently retired Finnish men and women [65.2 (SD 1.1) years, 83% women] took part to the study. The participants were randomized into intervention (n = 117) and control (n = 114) groups. The intervention group members used a commercial activity tracker (Polar Loop 2, Polar, Kempele, Finland) with a daily activity goal and inactivity alerts every day for 12 months. Controls received no intervention. Secondary health outcomes included body weight, fat mass, fat free mass, waist circumference, blood pressure, indicators of glucose and lipid metabolisms, and high-sensitivity C-reactive protein, and they were measured at baseline and at 12-months end point. Hierarchical linear mixed models were used to examine the differences between the groups over time, and no differences in the mean changes of the body composition and cardiometabolic health indicators between the groups were found (group*time interaction >0.20 for all measures). Fat free mass, waist circumference, blood pressure, and low density lipoprotein levels decreased in both groups over the 12 months. These findings state that 1-year daily use of commercial activity tracker does not induce different cardiometabolic health effects when compared to the non-user controls among general population of recent retirees.

## Introduction

Because the baby boomers are already at their retirement age, there is need to find feasible ways to support maintenance of health and functioning of this large ageing population. Compelling evidence shows that participation in regular physical activity reduces the risk of many chronic diseases (2018 Physical Activity Guidelines Advisory Committee, 2018) and mobility limitations in old age (Pahor et al., 2014). However, majority of older adults do not meet the physical activity recommendations (Evenson et al., 2012; Wullems et al., 2016). In addition, older adults are the most sedentary age group (Healy et al., 2011), further underlining the fact that effective interventions to promote physical activity among older adults are needed. Traditional intervention methods, such as face-to-face group/individual counselling or training sessions have shown to have the potential to be effective at increasing physical activity of older adults (Zubala et al., 2017). However, these methods usually require time and resources hampering their implementation as preventive programs at large scale. The more scalable and low-cost e-health intervention strategies utilizing devices and programs have been emerged (Muellmann et al., 2018). Consequently, more high quality studies of the use of technology-based methods to promote physical activity among older population are needed (Stockwell et al., 2019).

Modern fitness and activity trackers have increased their popularity among consumers but also as an intervention method because wearable technology can be harnessed with evidence-based behavioral change techniques, such as self-monitoring, goal setting and feedback, without face-to-face contact (Chia et al., 2019; Laranjo et al., 2020). According to the recent studies among older adults, short-term physical activity interventions utilizing activity trackers can increase physical activity (Coughlin and Stewart, 2016; Jonkman et al., 2018; Stockwell et al., 2019), and decrease body weight (Wijsman et al., 2013; Ashe et al., 2015; Cadmus-Bertram et al., 2015; Lyons et al., 2017; Vandelanotte et al., 2018; Ballin et al., 2020) and systolic blood pressure levels (Wijsman et al., 2013; Ashe et al., 2015). In addition, Wijsman et al. found that the use of activity monitors and digital coaching (DirectLife program, Philips) for 12 weeks increased daily moderate-to-vigorous physical activity by 11 min among inactive 60–70 years old adults and further improved their metabolic health in terms of decreased waist circumference and fat percentage, and improved glucose metabolism when the intervention group was compared to the control group not using the program (Wijsman et al., 2013). Consumer-wearable activity trackers have also been found to increase physical activity and to be associated with improvements in cardiometabolic health, including waist circumference, systolic blood pressure, and low-density lipoprotein cholesterol concentration in middle-aged and older patients with chronic diseases (Franssen et al., 2020). A 2-years pedometer-based intervention prevented decrease in daily steps but did not improve cardiometabolic health in over 60 years old patients with prediabetes or type 2 diabetes (Rossen et al., 2021). However, the long-term effects of activity tracker based physical activity interventions on cardiometabolic health outcomes among older adults are still unknown (Coughlin and Stewart, 2016; Stockwell et al., 2019).

We conducted a 12-months-long physical activity intervention that primarily aimed to increase total physical activity among recent retirees with the daily use of a commercial activity tracker (REACT, NCT03320746). The intervention was based on observational findings suggesting that older adults are prone to change physical activity behavior especially in the retirement transition (Barnett et al., 2012; Stenholm et al., 2016). Results regarding the primary outcome, daily physical activity, has been reported earlier (Leskinen et al., 2021). We did not find long-term intervention effect on daily total physical activity although a tendency for increased levels in total and light physical activity was seen during the first 6 months of the intervention. The changes were not however different from that of controls (Leskinen et al., 2021). Because the previous literature suggest that the retirement transition is also related to negative changes in health, such as weight gain, and increased blood pressure and lipid levels (Pedron et al., 2020) as well as increased sedentary behavior (Leskinen et al., 2018; Suorsa et al., 2020), this time-window is of importance for the prevention of cardiovascular disease risk factors (Xue et al., 2020), and thus should be further evaluated. Therefore, in the present study, we examined the effects of the REACT physical activity intervention on body composition and cardiometabolic health, the secondary outcomes of the trial.

## Methods

### Study Population

The REACT trial (NCT03320746) has been approved by the ethics committee of Hospital District of Southwest Finland (107/1801/2017). The study population of the trial included 231 Finnish public sector employees who had retired between January 2016 and December 2018. The recruitment and randomization protocols are reported elsewhere (Leskinen et al., 2021). After the randomization was completed, the initialized activity trackers along with detailed instructions on how to use the tracker and the manufacture’s web-based program were mailed to intervention group members. The control group members were informed of their allocation by e-mail and no further instructions were given.

The intervention participants (n = 117) were requested to wear commercial activity trackers (Polar Loop 2, Polar, Kempele, Finland) on their non-dominant wrists every day and night for 12 months. The control group members (n = 114) were requested to abstain from the use of any type of activity trackers during the 12-months follow-up. Overall, four participants discontinued intervention due to discomfort of the tracker and one control group participant discontinued the follow-up due to health issues.

### Intervention

The intervention content is described in detail elsewhere (Leskinen et al., 2021). Briefly, the intervention participants were instructed to aim to reach the daily activity goal provided by the tracker every day for 12 months. The daily activity goal, shown on the tracker’s display, initially corresponded for example to ∼1h/day of jogging, or ∼2 h/day of walking, or ∼7 h/day household activities, or a combination of activities at different intensities. The activity goals were pre-set by the trackers’ manufacturer and sensitive to user’s gender, age and typical daily activities. During each day, the tracker provided an instant view on how much activity was still needed to reach the goal and gave an inactivity alert if the user had an uninterrupted non-movement time for 55 min. No further counselling or guidance on how to achieve the daily activity goal was given to the participants. Upon fulfillment of the daily goal, the tracker congratulated the user. Once a week the intervention participants uploaded the activity data to their personalized accounts in the Polar’s web-based program, which then displayed the daily, weekly, and monthly patterns of physical activity behavior and gave the user feedback on activity benefits. The information was also used to follow the adherence and achievement of the daily activity goal. A higher daily activity goal was suggested by the researcher if the participant frequently exceeded the former goal.

### Body Composition and Metabolic Health Related Outcomes

The body composition and metabolic health related outcomes were measured during the baseline and at 12-months clinical visits at the University from all participants. A study nurse measured systolic and diastolic blood pressure, body height, waist circumference, and body composition by direct segmental multi-frequency bioelectrical impedance analysis method (InBodyJ10, Seoul, Korea) after the participant had had a 2-h fast. Blood pressure measurements were conducted in a seated positions with two repeated measurements. Waist circumference was measured two times between the lower rib and iliac crest and recorded to the nearest 0.1 cm. For both measures, the average values were used in the analysis. Body composition measures included body weight (kg), body mass index (BMI, kg/m^2^), body fat mass (kg), fat free mass (kg), and percent of body fat (%).

The 10-h fasting plasma and blood samples were taken and analyzed in the Turku University Hospitals’ laboratory division (Tykslab, Turku, Finland) at baseline and 12-months time point from all participants. The participants were requested to give the samples in the laboratory within 2 weeks from the clinical visits. Cardiometabolic health indicators included: *hemoglobin A1c* measured with the immunoturbidimetry by Cobas 6,000 c 501automatic analyzer (Roche Diagnostics GmbH, Mannheim, Germany); *fasting plasma glucose* level with the hexokinase method, *triglycerides* with the GPO-PAP method, *total cholesterol* with the CHOD-PAP method, *high-density lipoprotein* (HDL) cholesterol and *low-density lipoprotein* (LDL) cholesterol with the Direct method all by Cobas 8,000 c 702 automatic analyzer (Roche Diagnostics GmbH, Mannheim, Germany); and *high-sensitivity C-reactive protein* (hs-CRP) measured from serum with immunonephelometry by ProSpec (Siemens Medical Solutions, United States). Tykslab is accredited according to SFS-EN ISO 15189:2013 standard by the Finnish Accreditation Service (FINAS).

These health outcomes were selected as they have been used in previous multicomponent studies utilizing activity trackers, were available, and physical activity have been shown to have an effect on these indicators among older adults (Chodzko-Zajko et al., 2009).

### Physical Activity Outcome

A wake time total physical activity was measured using a wrist-worn triaxial ActiGraph wGT3X-BT, which participants wore for eight days and seven nights at baseline and at 3, 6 and 12-months follow-up points. The accelerometer data were analyzed using the R-package GGIR version 1.7–1 (Migueles et al., 2019) with a data procedure reported elsewhere (Leskinen et al., 2021).

### Background Characteristics

Date of birth, gender, and pre-retirement occupation were derived from pension institute’s register. Other baseline characteristics, including retirement age, current smoking status, alcohol risk use, and doctor-diagnosed diabetes, were assessed by the web-based questionnaire as reported earlier (Leskinen et al., 2021).

### Statistical Methods

Baseline characteristics of the study participants are presented as numbers and percentages for categorical variables. Linear mixed models for repeated measurements were used to examine the mean change differences between the groups. Compound symmetry for longitudinal covariance was used. The model included intervention group as a between-factor, time as a within-factor, and the group by time interaction. All results are shown as mean estimates and their 95% confidence intervals (CIs). Pearson correlation was used to study the associations between the 12-months change in the primary and secondary outcomes. *p*-values less than 0.05 (two-tailed) were considered as statistically significant. The SAS Software 9.4 (or later) was used for statistical analyses (SAS Institute Inc., Cary, NC).

## Results

Baseline characteristics of the intervention and control group participants are presented in Table 1.

**TABLE 1:** Baseline characteristics for the intervention and control group participants.

Characteristics	Intervention (n = 117)	Control (n = 114)
	n (%)	n (%)
**Age, mean (SD) years**	65.2 (1.0)	65.2 (1.1)
**Gender**	—	—
Women	96 (82.0)	95 (83.3)
Men	21 (18.0)	19 (16.7)
**Occupational status**	—	—
High	47 (40.2)	41 (36.0)
Intermediate	35 (29.9)	28 (24.5)
Low	35 (29.9)	45 (39.5)
**Body mass index**	—	—
Under/normal weight	38 (32.5)	43^a^ (37.7)
Overweight	43 (36.7)	45 (39.5)
Obese	36 (30.7)	26 (22.8)
**Current smoking**	—	—
No	113 (96.5)	109 (96.5)
Yes	4 (3.5)	4 (3.5)
**Alcohol risk use**	—	—
No	111 (94.9)	108 (94.7)
Yes	6 (5.1)	6 (5.1)
**Hypertension#**	—	—
No	59 (50.4)	52 (45.6)
Yes	58 (49.6)	62 (54.4)
**Diagnosed diabetes**	—	—
No	109 (94)	106 (93.8)
Yes	7 (6)	7 (6.2)
** Daily total physical activity, mean (SD) min**	279.1 (89.9)	271.1 (91.8)

SD = standard deviation.

a Including four participants with underweight.

#Baseline blood pressure ≥135/85.

**FIGURE 1:**
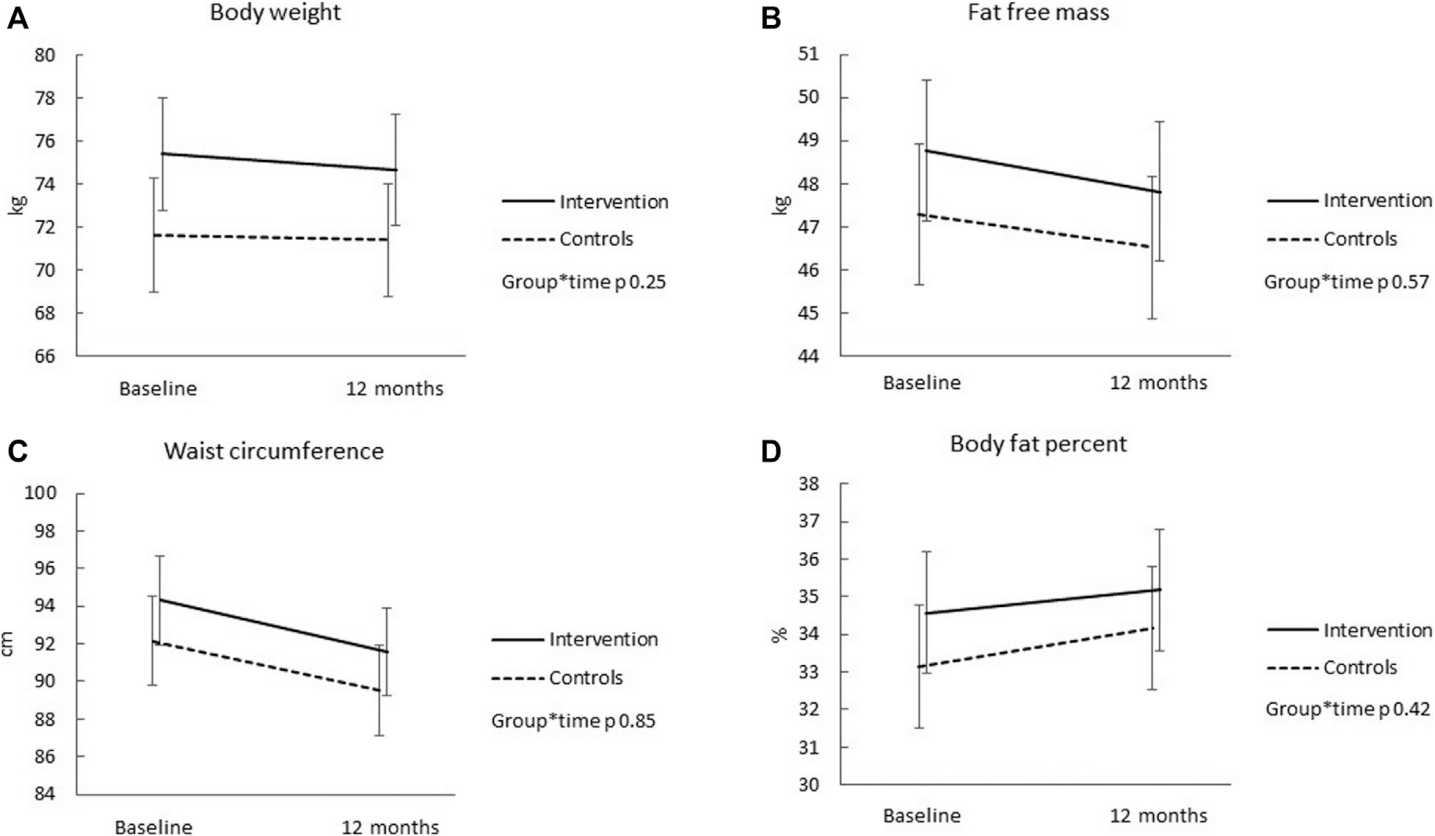
The change in body weight **(A)**, fat free mass **(B)**, waist circumference **(C)**, and body fat percent **(D)** over the 12 months for the intervention (solid line) and control (dotted line) groups. Results are expressed as means and 95% CIs based on mixed models.

**FIGURE 2:**
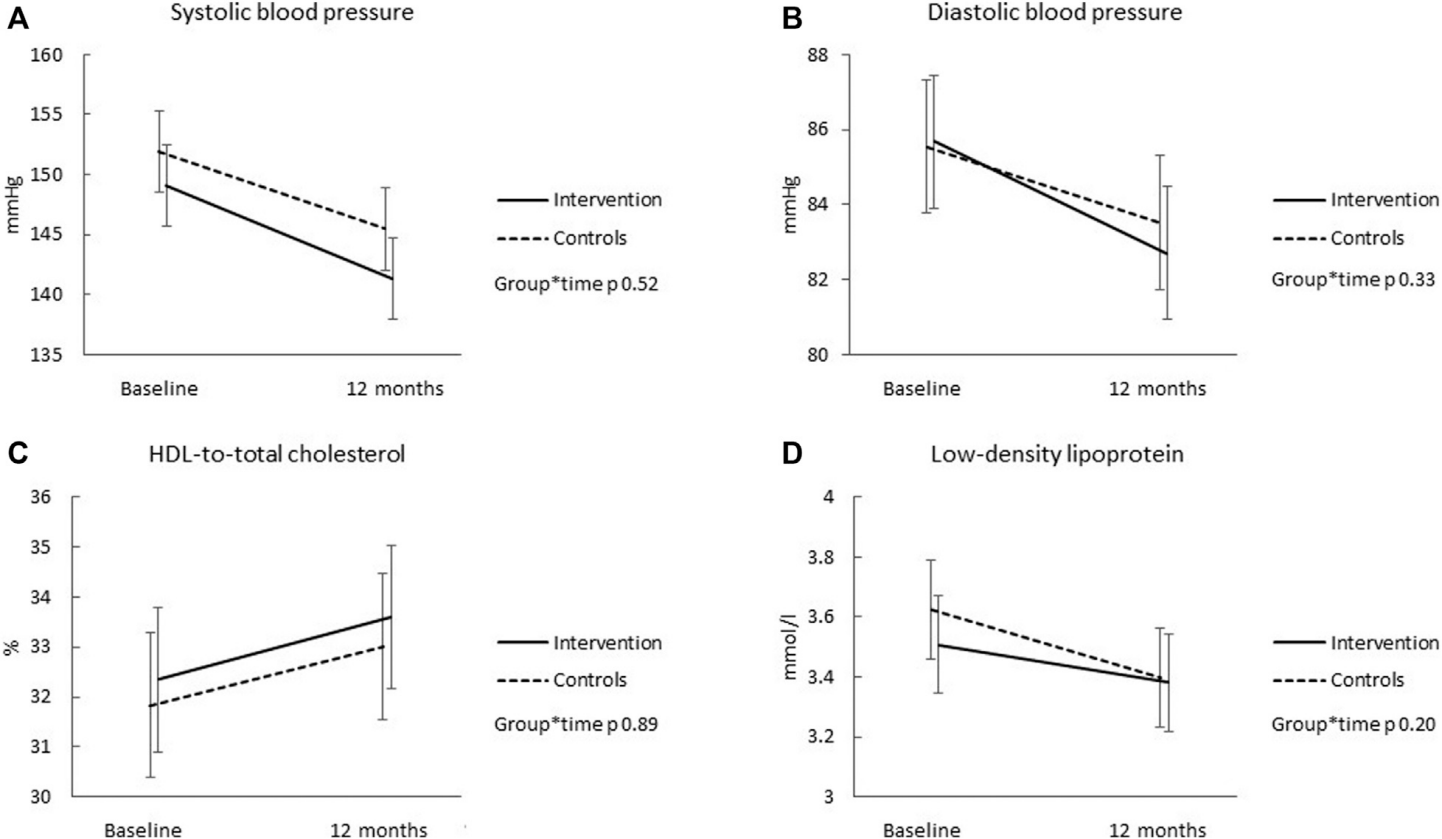
The change in systolic blood pressure **(A)**, diastolic blood pressure **(B)**, HDL-to-total cholesterol ratio **(C)**, and low-density lipoprotein levels **(D)** over the 12 months for the intervention (solid line) and control (dotted line) groups. Results are expressed as means and 95% CIs based on mixed models.

Details of the group comparisons for all body composition and cardiometabolic outcomes are presented in Supplementary Tables S1, S2. Figures 1, 2 illustrate the results, respectively. Over the 12 months, there were no significant differences in the mean changes in body composition and cardiometabolic health indicators between the groups (group*time interaction >0.20 for all outcomes). The body weight decreased in the intervention group over the 12 months (−0.7 kg, 95% CI −.3 to −0.2); but no significant change was observed in the control group (−0.7 kg, 95% CI −0.8 to 0.3) (Figure 1A). Both groups decreased waist circumference (−2.7 cm, 95% CI −3.4 to −2.1 for the intervention, and −2.6 cm, 95% CI −3.3 to −2.0 for the control group) (Figure 1B) and fat free mass (−1.0 kg, 95% CI −1.4 to −0.5 for the intervention, and −0.8 kg, 95% CI −1.2 to −0.3 for the control group) (Figure 1C), but only control group increased body fat percent (0.6 kg, 95% CI −0.1 to 1.3 for the intervention, and 1.0 kg, 95% CI 0.3 to 1.7 for the control group) (Figure 1D). Systolic blood pressure decreased significantly in the intervention (−7.8 mmHg, 95% CI −10.7 to −4.9) and control (−6.5 mmHg, 95% CI −9.4 to −3.6) groups (Figure 2A). Similarly, diastolic blood pressure decreased in both groups (−3.0 mmHg, 95% CI −4.3 to −1.6 for the intervention, and −2.0 mmHg, 95% CI −3.4 to −0.7 for the controls) (Figure 2B). There was also enhancement in the lipid profile among both groups over the 12 months (Figures 2C,D). The change in daily total physical activity (primary outcome) over the 12 months showed mild, but significant correlations with the change in systolic (r = -0.16, *p* = 0.0148) and diastolic blood pressure (r = 0.22, *p* = 0.0014), but not with any other secondary health related outcomes (results not shown).

## Discussion

The REACT trial examined the effect of a 12-months commercial activity tracker based intervention primarily on physical activity behavior and secondarily on other health-related outcomes among recently retired older adults. The present secondary analyses showed no differences in the changes in body composition and cardiometabolic health indicators between the groups over time.

Previous short-term multicomponent physical activity interventions utilizing activity trackers as part of their interventions have succeeded to increase physical activity among individuals having low levels of physical activity or overweight at baseline (Coughlin and Stewart, 2016; Jonkman et al., 2018; Stockwell et al., 2019). Our long-term intervention among general population of recent retirees showed that the use of commercial activity tracker as a stand-alone intervention method did not induce significant changes in daily total physical activity over the 12 months (Leskinen et al., 2021). This result likely explains why no intervention effect was observed in any of the body composition and cardiometabolic health outcomes included in the secondary analysis. The previous digital exercise interventions targeted to sedentary older adults having overweight or chronic diseases have increased mostly moderate-to-vigorous physical activity and resulted in improved metabolic health in the short-term (Wijsman et al., 2013; Ashe et al., 2015; Ballin et al., 2020; Franssen et al., 2020). However, apart from our long-term RCT design, we were unable to examine the short-term effects, because we did not measure body composition nor cardiometabolic outcomes during the intervention. Due to lack of changes in physical activity behavior and especially in the levels of moderate-to-vigorous physical activity over 12 months, our findings are in line with 2-years pedometer-based intervention in which no significant increase in daily steps nor improved metabolic health was observed (Rossen et al., 2021). It is likely that changes in moderate-to-vigorous physical activity are needed to have more marked changes in metabolic health. Therefore, our findings may present aging-related changes or they may be explained by changes in other health behaviors, such as changes in dietary intake, which were not controlled in the study. The controls were not instructed (nor restricted) to engage in physical activity but for example the baseline health measurements may have motivated participants to change their physical activity behavior. The participants of this study were mainly healthy and active older women, which may limit the comparison as well as the generalizability of our findings to other populations.

In conclusion, among general population of recent retirees, the 1-year use of commercial activity trackers with daily activity goal did not induce different cardiometabolic health effects compared to the non-user controls. Alternative methods are therefore needed to promote especially moderate-to-vigorous physical activity and health among retired older adults.

## Data Availability

Anonymized partial datasets of the REACT trial are available by application for bona fide researchers with an established scientific record and bona fide organizations.
