# Supplementary material for: The Effect of Commercial Activity Tracker Based Physical Activity Intervention on Body Composition and Cardiometabolic Health Among Recent Retirees
Source: Front Aging. 2021 Oct 29;2:757080. doi: 10.3389/fragi.2021.757080 (PMC9261302; doi:10.3389/fragi.2021.757080)
Supplement: Supplementary file 1 [file Table1.DOCX]

Supplementary table 1. Change in body composition measures over 12 months in intervention and control groups. Results are as means and their 95% CIs based on mixed models.

|  | **Intervention** |  |  | **Control** |  |  | **P-values** |  |  |
| --- | --- | --- | --- | --- | --- | --- | --- | --- | --- |
|  | Mean | 95% CI |  | Mean | 95% CI |  | Group | Time | Group*Time |
|  |  |  |  |  |  |  |  |  |  |
| **BMI (kg/m2)** |  |  |  |  |  |  | 0.207 | 0.164 | 0.369 |
| Baseline | 27.6 | 26.8 | 28.5 | 26.8 | 25.9 | 27.7 |  |  |  |
| Change at 12 months | -0.2 | -0.4 | 0.04 | -0.04 | -0.3 | 0.2 |  |  |  |
| **Body weight (kg)** |  |  |  |  |  |  | 0.061 | 0.021 | 0.247 |
| Baseline | 75.4 | 72.8 | 78.0 | 71.6 | 69.0 | 74.3 |  |  |  |
| Change at 12 months | -0.7 | -1.3 | -0.2 | -0.2 | -0.8 | 0.3 |  |  |  |
| **Body fat mass (kg)** |  |  |  |  |  |  | 0.099 | 0.103 | 0.562 |
| Baseline | 26.6 | 24.8 | 28.4 | 24.4 | 22.5 | 26.2 |  |  |  |
| Change at 12 months | 0.3 | -0.4 | 0.9 | 0.5 | -0.1 | 1.2 |  |  |  |
| **Fat free mass (kg)** |  |  |  |  |  |  | 0.233 | <.0001 | 0.574 |
| Baseline | 48.8 | 47.1 | 50.4 | 47.3 | 45.6 | 48.9 |  |  |  |
| Change at 12 months | -1.0 | -1.4 | -0.5 | -0.8 | -1.2 | -0.3 |  |  |  |
| **Body fat percent (%)** |  |  |  |  |  |  | 0.285 | 0.002 | 0.417 |
| Baseline | 34.6 | 33.0 | 36.2 | 33.2 | 31.5 | 34.8 |  |  |  |
| Change at 12 months | 0.6 | -0.1 | 1.3 | 1.0 | 0.3 | 1.7 |  |  |  |
| **Waist circumference (cm)** |  |  |  |  |  |  | 0.214 | <.0001 | 0.852 |
| Baseline | 94.3 | 92.0 | 96.6 | 92.2 | 89.8 | 94.5 |  |  |  |
| Change at 12 months | -2.7 | -3.4 | -2.1 | -2.6 | -3.3 | -2.0 |  |  |  |
|  |  |  |  |  |  |  |  |  |  |

CI=confidential interval, BMI=body mass index
