# Supplementary material for: The Effect of Commercial Activity Tracker Based Physical Activity Intervention on Body Composition and Cardiometabolic Health Among Recent Retirees
Source: Front Aging. 2021 Oct 29;2:757080. doi: 10.3389/fragi.2021.757080 (PMC9261302; doi:10.3389/fragi.2021.757080)
Supplement: Supplementary file 2 [file Table2.DOCX]

Supplementary table 2. Change in cardiometabolic health indicators over 12 months in intervention and control groups. Results are as means and their 95% CIs based on mixed models.

|  | **Intervention** |  |  | **Control** |  |  | **P-values** |  |  |
| --- | --- | --- | --- | --- | --- | --- | --- | --- | --- |
|  | Mean | 95% CI |  | Mean | 95% CI |  | Group | Time | Group*Time |
|  |  |  |  |  |  |  |  |  |  |
| **SBP (mmHg)** |  |  |  |  |  |  | 0.118 | <.0001 | 0.522 |
| Baseline | 149.1 | 145.7 | 152.5 | 151.9 | 148.5 | 155.3 |  |  |  |
| Change at 12 months | -7.8 | -10.7 | -4.9 | -6.5 | -9.4 | -3.6 |  |  |  |
| **DBP (mmHg)** |  |  |  |  |  |  | 0.771 | <.0001 | 0.328 |
| Baseline | 85.7 | 83.9 | 87.4 | 85.5 | 83.8 | 87.3 |  |  |  |
| Change at 12 months | -3.0 | -4.3 | -1.6 | -2.0 | -3.4 | -0.7 |  |  |  |
| **HbA1c (mmol/l)** |  |  |  |  |  |  | 0.319 | <.0001 | 0.941 |
| Baseline | 36.7 | 35.9 | 39.0 | 36.1 | 35.3 | 37.0 |  |  |  |
| Change at 12 months | 1.5 | 0.8 | 2.1 | 1.5 | 0.9 | 2.0 |  |  |  |
| **FPG (mmol/l)** |  |  |  |  |  |  | 0.691 | 0.181 | 0.35 |
| Baseline | 5.7 | 5.6 | 5.8 | 5.7 | 5.6 | 5.8 |  |  |  |
| Change at 12 months | -0.01 | -0.1 | 0.1 | -0.1 | -0.2 | 0.01 |  |  |  |
| **TG (mmol/l)** |  |  |  |  |  |  | 0.947 | 0.0006 | 0.225 |
| Baseline | 1.3 | 1.2 | 1.4 | 1.2 | 1.1 | 1.3 |  |  |  |
| Change at 12 months | -0.1 | -0.2 | -0.1 | -0.1 | -0.1 | 0.01 |  |  |  |
| **TC (mmol/l)** |  |  |  |  |  |  | 0.349 | 0.0003 | 0.209 |
| Baseline | 5.4 | 5.2 | 5.6 | 5.6 | 5.4 | 5.7 |  |  |  |
| Change at 12 months | -0.1 | -0.2 | 0.02 | -0.2 | -0.3 | -0.1 |  |  |  |
| **HDL (mmol/l)** |  |  |  |  |  |  | 0.825 | 0.651 | 0.430 |
| Baseline | 1.7 | 1.6 | 1.8 | 1.7 | 1.7 | 1.8 |  |  |  |
| Change at 12 months | 0.02 | -0.02 | 0.05 | -0.01 | -0.04 | 0.03 |  |  |  |
| **HDL/TC (%)** |  |  |  |  |  |  | 0.582 | <.0001 | 0.90 |
| Baseline | 32.3 | 30.9 | 33.8 | 31.8 | 30.4 | 33.3 |  |  |  |
| Change at 12 months | 1.3 | 0.4 | 2.1 | 1.2 | 0.3 | 2.0 |  |  |  |
| **LDL (mmol/l)** |  |  |  |  |  |  | 0.552 | <.0001 | 0.20 |
| Baseline | 3.5 | 3.3 | 3.7 | 3.6 | 3.5 | 3.8 |  |  |  |
| Change at 12 months | -0.1 | -0.2 | -0.02 | -0.2 | -0.3 | -0.1 |  |  |  |
| **hs-CRP (mg/l)** |  |  |  |  |  |  | 0.197 | 0.746 | 0.816 |
| Baseline | 1.8 | 1.2 | 2.4 | 2.3 | 1.7 | 3.0 |  |  |  |
| Change at 12 months | -0.03 | -0.8 | 0.8 | -0.2 | -0.9 | 0.6 |  |  |  |

CI=confidential interval, SBP=systolic blood pressure, DBP=diastolic blood pressure, HbA1c= hemoglobin A1c, FGP=fasting plasma glucose, TG=triglycerides, TC=total cholesterol, HDL=high-density lipoprotein, LDL=low-density lipoprotein, hs-CRP= high-sensitivity C-reactive protein
